# Stability in the feeding practices and styles of low-income mothers: questionnaire and observational analyses

**DOI:** 10.1186/s12966-018-0656-6

**Published:** 2018-03-23

**Authors:** Karina Silva Garcia, Thomas G. Power, Ashley D. Beck, Jennifer Orlet Fisher, L. Suzanne Goodell, Susan L. Johnson, Teresia M. O’Connor, Sheryl O. Hughes

**Affiliations:** 1 Department of Human Development, Washington State University, PO Box 644852, Pullman, WA 99164-4852 USA; 2 Center for Obesity Research and Education, Temple University, 3223 N. Broad Street, Suite 175, Philadelphia, PA 19140 USA; 3 Department of Food, Bioprocessing, and Nutrition Sciences, North Carolina State University, 100 Schaub Hall, Campus Box 7624, Raleigh, NC 27695-7624 USA; 4 Children’s Eating Laboratory, Department of Pediatrics, University of Colorado Denver, Building 500, Box F561, 13001 East 17th Place, Aurora, CO 80045 USA; 5 USDA/ARS Children’s Nutrition Research Center, Department of Pediatrics, Baylor College of Medicine, 1100 Bates, Houston, TX 77030-2600 USA

**Keywords:** Mothers, Feeding practices, Feeding styles

## Abstract

**Background:**

During the last two decades, researchers have devoted considerable attention to the role of maternal feeding behaviors, practices, and styles in the development of obesity in young children. Little is known, however, about the consistency of maternal feeding across settings and time. The purpose of this paper was to provide data on this issue by examining the consistency of observed maternal feeding behavior across multiple eating occasions, as well as examine the consistency of observed and self-reported maternal feeding behavior across 18 months.

**Methods:**

Videotapes from two studies of low-income mothers and their preschool children were coded for feeding practices, dimensions, and styles: a study of 137 low-income, African American and Latina mothers and their children observed during three meals in their homes over a two to three week period, and a study of 138 low-income, Latina mothers observed during a buffet meal in a laboratory setting on two separate occasions 18 months apart. Videotapes from both studies were coded for a wide range of maternal feeding behaviors and strategies. Mothers in the second study also completed three validated, self-report questionnaires on their feeding practices and styles.

**Results:**

Overall, both observed and self-reported feeding practices and styles showed only moderate levels of stability across meals and over time. Maternal attempts to regulate children’s eating showed more stability across meals and over time than the content of general mealtime conversation. Also, greater stability was found in *what* mothers were trying to get their children to do during the meals than in the *strategies* they used to influence child behavior. Self-reports of feeding showed greater stability over time than observational measures. Across meals and across 18 months, the stability of general feeding *styl*es was between 40% and 50%.

**Conclusions:**

The findings demonstrate that maternal feeding behavior was only moderately stable across meals and over time—that is, feeding behavior varied considerably across situations. The lack of high levels of consistency in feeding behavior suggests that situational factors may play a major role in influencing maternal behavior as well. Family-focused childhood obesity programs should focus not only on helping parents change their feeding practices and styles, but also focus on increasing parents’ sensitivity to situational factors that affect their approach to feeding their children.

## Background

Over the last 20 years, researchers have examined extensively the role of food parenting practices and styles on the development of preschool children’s eating behavior and childhood obesity [1, 2]. For example, parental restriction has been associated with poor regulation of caloric intake and the consumption of junk food, sweets, and other unhealthy snacks; parental pressure to eat has been associated with poor food intake and picky eating; and parental modeling of healthy consumption has been associated with children’s healthy eating [3]. Feeding style is important as well. Numerous studies show, for example, that the indulgent feeding style is associated with greater child intake of unhealthy foods and positively associated with childhood obesity [4].

The primary approach to measuring parental feeding in these studies has been the use of parent or child self-report interviews or questionnaires. For example, in a systematic review published in 2013 [5], 71 different parent or child self-report measures of parental feeding practices and styles were identified. In contrast, several researchers have conducted behavioral observations of parental feeding practices in home or lab settings. The number of such studies, however, is small. For example, in a recent review of observational studies of preschool children [6], only 13 studies were identified. This reliance on self-report measures is concerning given that participants may not accurately remember how often they engaged in specific behaviors, may not accurately average across multiple occurrences of a behavior, or may not be consciously aware of the behavior being assessed [7].

A critical question regarding the measurement of parental feeding practices and styles is the degree to which these behaviors are consistent across situations and across time. For parental feeding practices and styles to influence child eating behavior and obesity risk, parents must have a relatively stable approach to feeding their child. If parental feeding styles and practices vary widely across time and situations, they would unlikely have a consistent impact on child eating behavior and health. Research on this issue is limited. *Self-reports* of feeding behavior appear to be stable over very short periods of time. For example, several well-validated feeding questionnaires report test-retest reliability values generally ranging from .65 to .91 over 1 to 2 weeks: e.g., the Child Feeding Questionnaire [8, 9], the Parental Feeding Style Questionnaire [10], the Caregiver’s Feeding Style Questionnaire [11], the Overt and Covert Control Questionnaire [12, 13], and the Comprehensive Feeding Practices Questionnaire [14, 15].

Fewer data have been reported on the longer-term stability of such measures. Although numerous longitudinal studies using parental feeding questionnaires have been conducted [1, 2], most publications do not report data on stability over time. However, two studies of the Child Feeding Questionnaire [8] showed relatively high levels of stability over a two-year period [16, 17]: pressure to eat (.64 to .83), restriction (.46 to .59), and monitoring (.23 to .31).

Although these correlations show short- and long-term stability in maternal self-reports of feeding practices, they may not necessarily reflect consistency in actual maternal *behavior.* Such correlations may reflect mothers attempting to appear consistent over time (for the test-retest studies, most mothers likely remembered how they filled out the questionnaire 1 to 2 weeks earlier) or, as argued by Mischel [18], consistency in self-reports more likely reflects individuals’ cognitive constructions of what their behavior is like (i.e., what kind of mother are they?) versus consistency in their actual behavior. In 1968, Mischel [18] reviewed research showing that although observed behaviors are rarely that consistent across situations, self-reports of behavior and global descriptions of behavior by others often are.

Subsequent research on the consistency of self-reports and behavioral observations led to several conclusions: 1) in many large-scale, classic studies, the cross-situational correlations between observed behaviors measuring underlying “traits” (e.g., honesty, extraversion, punctuality) usually correlated with one another around r = .20 accounting for less than 5% of the variance thereby demonstrating the situation-specific nature of human behavior [19]; 2) aggregation across multiple observational measures with low intercorrelations can yield acceptable levels of cross-situational stability [20] (for example, with nine measures of a construct with a mean intercorrelation of .21, the equation for the standardized alpha yields a value of .70); 3) temporal stability (i.e., consistency across identical situations) is greater than cross-situational consistency [19]; 4) the more similar the situations, the higher the cross-situational correlations [21]; and 5) higher-order constructs such as behavior patterns or patterns of behavioral contingencies show higher stability across situations than individual behaviors alone [22].

The purpose of this paper was to examine consistency of maternal feeding behavior in two samples of low-income, mothers of preschool children in a large urban area in the U.S. The first sample consisted of mothers [23] observed across three separate dinners in their homes over a two to three week period. The second sample consisted of mothers [24] observed feeding their child during a buffet meal in a laboratory setting two times, 18 months apart. The mothers in the second study completed several widely used feeding questionnaires at both time points as well, allowing us to: 1) examine stability in observed feeding practices and styles across three separate meals and across an 18-month period; 2) examine stability in self-reported feeding practices across an 18-month period; and 3) test several specific hypotheses about stability and change.

Based upon the literature reviewed above, it was predicted that: 1) mothers would show moderate levels of stability in their feeding practices and styles across meals within a time point; 2) mothers would show greater levels of stability of observed behavior across meals than across 18 months; 3) self-reports of feeding would be more stable over 18 months than observed feeding variables; 4) maternal reports of pressure to eat and restriction would show greater levels of stability over an 18 month period than maternal monitoring [16, 17]; and 5) for both the observed and self-report measures, higher-order measures of feeding dimensions and styles (i.e., measures that aggregate across multiple individual feeding behaviors) would show higher levels of stability over time and over meals than individual feeding practices.

## Methods

### Participants

Participants came from two separate studies—there was no overlap in participants between the two studies.

#### Study 1

The videotapes coded for this study came from a larger study of parent-child interactions at dinner in low-income families from a large urban area in the southern U.S. [23]. In this study of 177 families, observers in the home coded parent-child interactions at three separate meals per family—the results of this live coding are reported in Hughes et al. [23]. Only 145 of these families were videotaped for later coding due to funding limitations. Of the 145 families, only 137 videotapes were codeable (e.g., for 8 videotapes the sound was inaudible, light conditions were problematic, etc.). Videotapes used in Study 1 included 131 families at Time 1, 135 at Time 2, and 129 at Time 3. Demographics are presented in Table 1. Chi Square analyses and t-tests revealed no significant differences between mothers with usable videotapes and mothers who were either not videotaped or whose tapes were uncodable.

**Table 1:** Characteristics of the Latina and African American Mothers of Preschoolers in Studies 1 and 2

	Study 1 (*n* = 137) Percentages	Study 2 (*n* = 138) Percentages
Ethnicity		
Latina	58	100
African-American	42	0
Education of Mother		
Less than High School Diploma	28	39
High School Diploma	25	24
Some College	32	33
College Graduate	10	4
Missing Data	5	0
Mother’s Place of Birth		
Born in the U.S.	50	17
Not Born in the U.S.	49	83
Missing Data	1	0
Marital Status of Mother		
Married	39	57
Divorced, Separated, Widowed, Other	20	30
Never Married	29	13
Missing	12	0
Employment of Mother		
Employed Part-time	22	17
Employed Full-time	29	4
Not Employed	44	76
Missing	5	3
Child Gender		
Female	50	46
Male	50	54
Child Weight Status		
Underweight (BMI < 5th percentile)	3	1
Healthy Weight (>5th to <85thBMI percentile)	57	49
Overweight (85^th^ percentile ≤ BMI < 95th percentile)	19	22
Obese (BMI ≥ 95th percentile)	20	28
Missing	1	0
Mother Weight Status		
Underweight (BMI ≤ 18.5 kg/m^2^)	0	1
Healthy Weight (18.5 kg/m^2^ < BMI < 25 kg/m^2^)	19	14
Overweight (25 kg/m^2^ ≤ BMI < 30 kg/m^2^)	28	34
Obese (BMI ≥ 30 kg/m^2^)	53	51
Age, Mean in Years (SD)		
Parent	32.28 (7.79)	32.03 (6.61)
Child	4.42 (0.66)	4.73 (0.45)

#### Study 2

Participants were 138, low-income Latina mothers and their children participating in a longitudinal study of feeding practices and child eating behavior [24] in the same urban area as Study 1. Time point 1 measures were conducted during a two-year period on a self-selected sample. Children with extensive diet restrictions or with behavioral or cognitive problems that would limit their ability to participate in the study tasks where excluded. A total of 187 mothers and their children participated at the first time point of the study. At 9 months, study staff contacted the mothers to maintain rapport and update contact information. Eighteen months after the first time point, data from 144 mothers and children were collected—138 had data on all of the variables for the present study. Comparison of mothers in the original sample with those who dropped out revealed two significant differences: 1) dropouts were less likely to have children who were obese, *X*^2^(2) = 5.88, *p* = .05, 17% versus 28%, and 2) dropouts were more likely to have been mothers who were born in the U.S., *X*^2^(1) = 5.36, *p* < .05, 33% versus 17%. Demographics are presented in Table 1.

### Procedures

As discussed in Hughes et al. [23, 24], mothers in both studies were recruited through Head Start Centers. Recruitment was ongoing during the course of the studies. Both studies had been approved by the Institutional Review Boards at the participating universities and the researchers received written consent from the mothers for their own and their child’s participation before any data were collected. In Study 1 [23], mothers and children were videotaped during three dinners in their home over a two to three week period. Mothers were told to do what they normally do at dinner time and to feed their child as they usually do. Eight-5 % of the observations took place at the dinner table. In about half the cases, the mother was eating together with the child at the table. Forty-three percent had a sibling joining the meal, but only behaviors between the study child and mother were coded for this study. The meals ranged from three to 46 min with a mean of 17 min (*SD* = 7 min).

In Study 2 [24], mothers came to a research center at two time points—18 months apart. Mothers visited the research center two times at each time point. Child care and transportation were provided as needed to attend the study visits. On the first day of each time point, mothers were videotaped with unobtrusive cameras while eating a buffet meal with their child in a laboratory setting. Mothers and children chose foods from a buffet including spaghetti, sauces, vegetables, fruit, and desserts. The mothers were told: “Please help yourself and your child to the food from this cart. It is buffet style so you and your child can have whatever you’d like.” The meals ranged in length from 11.47 min to 57.80 min (M = 36.08, SD = 8.64) at the first time point and from 12.04 min to 48.56 min (M = 36.81, SD = 7.28) at second time point. Mothers completed questionnaires while their child was involved in assessments in a separate room. Bilingual research assistants were available to answer any questions.

### Self-report feeding measures (study 2 only)

#### Child Feeding Questionnaire (CFQ) [8]

The CFQ measures three feeding practices (restriction, pressure to eat, and monitoring). This measure has been used and validated in low-income samples [25]. Although test-retest reliability was not assessed in the instrument development sample, Nowika and colleagues [9] test-retest reliability over a two-week period in a Swedish sample was: restriction (*r* = .73), pressure to eat (*r* = .76), and monitoring (*r* = .56). The coefficient alphas for these subscales in the current study are presented in Table 4.

#### Caregiver’s Feeding Styles Questionnaire (CFSQ) [11]

The CFSQ measures parental feeding styles by assessing two feeding dimensions: demandingness and responsiveness. Demandingness is the overall degree that the mother encourages her child to eat; responsiveness is how sensitive she is to her child’s individual needs during feeding. Median splits on these dimensions are used to assign mothers to one of four feeding styles: authoritative (high demandingness/high responsiveness), authoritarian (high demandingness/low responsiveness), indulgent (low demandingness/high responsiveness), and uninvolved (low demandingness/low responsiveness). Validity of the questionnaire has been demonstrated in a large number of studies showing that in low-income samples, the indulgent style is most often associated with childhood obesity [4]. Test-retest reliability over a 1- to 2-week period was 0.85 for demandingness and 0.82 for responsiveness [11].

#### Comprehensive Feeding Practices Questionnaire (CFPQ) [14]

The CFPQ measures 12 feeding practices: child control, emotion regulation, encourage balance and variety, environment, food as reward, involvement, modeling, monitoring, pressure, restriction for health, restriction for weight control, and teaching about nutrition. The instrument has been validated in low-income samples [26, 27]. Although test-retest reliability was not assessed in the instrument development sample, Warkentin and colleagues [15] found that test-retest reliability over a two-week period in a Brazilian sample ranged from .48 to .81. The coefficient alphas for these subscales in the current study are presented in Table 4.

### Videotape coding

Videotapes from both studies were coded using the same procedures and coding system. All videotapes were transcribed in the language used by the mother and child. Using the Noldus Observer software (Observer XT, Noldus Information Technology, Wageningen, Netherlands), videotapes were coded by B.A. level employees blind to the purposes of the study. Coders were trained on videos collected as part of pilot research for these studies and coding did not begin until coders had demonstrated acceptable levels of reliability on several videos (kappas > .80). All training was conducted by the most experienced research assistant in collaboration with one of the authors. One quarter of the videotapes were coded independently by a second bilingual observer to assess inter-observer agreement. Interobserver agreement was assessed throughout the duration of the coding process. The coders were unaware of which observations had been selected for reliability assessment. Agreement was assessed with Cohen’s kappa [28].

Employing event coding, all maternal and child verbalizations, along with a number of nonverbal behaviors, were coded with a system adapted from Baumrind and Black [29] and Cousins, Power, and Olvera [30]. All maternal attempts to influence child behavior and child attempts to influence maternal behavior were coded (i.e., *influence attempts*), along with all other verbalizations between the mother and child (i.e., *non-influence attempts*). Data on only maternal influence attempts, non-influence attempts, and nonverbal behaviors were used in the current analyses (data on child behaviors are not reported here). Only maternal influence attempts were coded in Study 2 (non-influence attempts were not coded in this study because of the time-intensive nature of the coding system).

The codes analyzed for the current paper are presented in Table 2. More detail on the coding system can be obtained from the last author. Kappa statistics for the various aspects of the coding system ranged from .72 to .86 (mean = .77). Because several of the codes occurred with too low frequency for analysis (i.e., made up 1% of all influence or non-influence attempts or less), they were dropped for analyses of individual behaviors. The codes dropped were: Praise, Demonstrates, all reward and punishment codes, and all internal cues reference codes.

**Table 2:** Codes Analyzed for Current Paper

I. Child Behaviors Mothers Were Trying to Influence (Desired Behaviors)--Frequencies (see text)	
A. Encourage Eating	
B. Encourage Child to Eat All the Food on the Plate	
C. Encourage Child to Eat a Different Food	
D. Discourage Eating	
E. Enforce/Teach Table Manners	
F. Teach Eating Skills	
G. Internal Cues Reference to Encourage Eating^a^	
H. Internal Cues Reference to Discourage Eating^a^	
I. Other Food Related Behaviors (e.g., pass food, help sibling serve food)	
J. Non Food-Related Behaviors (e.g., discourage TV watching, be nice to sibling)	
II. Maternal Verbal Strategies used to Influence Child Behavior—Proportions (see text)	
A. Hint/Acknowledge	
B. Enthusiastic Modeling	
C. Question/Suggestion	
D. Praise^a^	
E. Reason/Instruct	
F. Unelaborated Command	
G. Verbal Pressure (e.g., “You *have* to eat it”)	
H. Promise Food Rewards^a^	
I. Threaten Food Punishments^a^	
J. Promise Non-Food Rewards^a^	
K. Threaten Non-Food Punishments^a^	
III. Maternal Non-Verbal Strategies to Influence Child Behavior—Proportions (see text)	
A. Moves Self Closer	
B. Moves Something Closer	
C. Points/Motions	
D. Demonstrates^a^	
E. Helps	
F. Spoon Feeds	
G. Physically Forces	
IV. Types of Non-Influence Attempts—Frequencies (see text)^b^	
A. References to Food Characteristics (e.g., appearance, smell, preparation)	
B. References to Other Food-Related Content (e.g., food placement, utensils)	
C. References to Target Child	
D. References to Mother	
E. References to Other People	
F. References to Non-Food Related Content (e.g., “It’s going to rain tomorrow.”)	
G. Clarification	

^a^Dropped from analyses of individual behaviors due to low frequency

^b^Coded in Study 1 only

### Data analysis

The first set of analyses tested the first part of Hypothesis 1: that maternal feeding *practices* would be moderately stable across the three meals in Study 1. First, the average correlation across each pair of meals (i.e., meal 1 with meal 2, meal 1 with meal 3, and meal 2 with meal 3) was calculated for each of the observed feeding variables in Table 2 that occurred with sufficient frequency for analysis. The variables for these analyses were the total frequencies of the various influence and non-influence attempt codes along with proportions for the various verbal and nonverbal strategies. Proportions were used for the strategy codes, because the number of influence attempts varied widely across meals and analyzing frequencies for the strategy codes would confound strategy use with the total frequency of influence attempts. The use of proportions for the strategy codes allowed us to identify a mother’s general *approach* to encouraging or discouraging child behavior controlling for how *often* she tried to influence child behavior. The numerators for these proportions were the frequencies that a particular code occurred and the dominator was the total frequency of influence attempts for that meal.

Next, to examine the stability of feeding *styles* across meals, measures of observed maternal demandingness and responsiveness during each meal were derived in a manner similar to that used in scoring the CFSQ. To calculate maternal demandingness, the sum of the frequency of all encouraging to eat items was calculated (i.e., Encourage Eating, Encourage Child to Eat All the Food on the Plate, and Internal Cues Reference to Encourage Eating). Because the meals differed widely in their length, the total frequency of the encouraging eating items was divided by the length of meal in minutes to create a rate per minute. This rate measure gave us a measure of demandingness that was not confounded by the length of the meals. To derive a measure of responsiveness, the sum of the child-centered verbal and nonverbal strategy codes (i.e., those feeding strategies labelled as such on the CFSQ) was divided by the total number of strategy codes from the CFSQ to yield the proportion of strategies that were child centered. The child-centered verbal codes were: Hint/Acknowledge, Enthusiastic Modeling, Question/Suggestion, Praise, and Reason/Instruct. Only one non-verbal child centered strategy was included for this analysis: Helps. The adult-centered strategies were: Unelaborated Commands, Verbal Pressure, Promise Food Rewards, Threaten Food Punishments, Promise Non-Food Rewards, Threaten Non-Food Punishments, Spoon Feeds, and Physically Forces. The other codes were not used in these analyses because they were behaviors *not* on the CFSQ (i.e., Moves Self Closer, Moves Something Closer, Points/Motions, and Demonstrates).

Once these dimension scores were calculated, the average correlation between the three meals for each dimension was examined. Median splits on observed demandingness and responsiveness were used to assign mothers to the four feeding styles as described above: authoritative, authoritarian, indulgent, and uninvolved. Finally, dichotomous variables were calculated for each feeding style. Mothers exhibiting a particular feeding style at a given meal were given a value of “1” for that feeding style variable; mothers not exhibiting that style were given a value of “0.” Then stability across meals was examined by calculating the mean correlation across the three meals for each dichotomous variable—the mathematical equivalent of the phi coefficient for 2 X 2 tables—a measure of association for nominal variables.

Based on Rosenthal’s [31] small, medium, large, and very large effect sizes, four levels of stability were defined: low (*r* = .10 to .29), moderate (*r* = .30 to .49), high (*r* = .50 to .69), and very high (*r* ≥ .70).

The second set of analyses examined Hypothesis 2: that greater stability would be observed across meals than across 18 months. This involved comparing the average correlations across the three meals (Study 1) to the correlations across 18 months (Study 2) for all of the observational feeding variables described above (i.e., practices, dimensions, and styles) with the exception of non-influence attempts. Unfortunately, we could not conduct statistical comparisons between the two studies because for each comparison we were comparing an average correlation across three meals to a single correlation over time. As an alternative, we determined that there was a difference in stability between the two studies if the difference between a pair of correlations was 0.20 or greater. We chose this cut-off because it was greater than the critical value for a correlation to be significantly greater than zero in each sample.

To test Hypothesis 3 (that self-reports would be more stable over 18 months than observed feeding variables), we compared the cross-time correlations for the self-report measures (i.e., the subscales of the CFQ and the CFPQ, the demandingness and responsiveness scores of the CFSQ, and the dichotomous variables corresponding to the four feeding styles from the CFSQ) to the correlations for the observational variables. Although direct comparison of many of the self-report and observational variables over time was not possible (because the constructs differed), we compared the mean correlations for those feeding practices for which we had both self-report and observational measures. For the observations, these variables were: encourage eating, eat all of food on the plate, discourage eating, and enthusiastic modeling. The corresponding variables for the self-reports were the two pressure to eat subscales, the three restriction subscales, and the modeling subscale. Because demandingness and responsiveness were calculated similarly for both the observational and self-report measures, the differences between the across-time correlations for the observational and self-report measures were examined with the modified Pearson-Filon statistic [32].

Hypothesis 4 (that maternal reports of monitoring would show less stability over time than pressure to eat and restriction) was tested comparing the cross-time correlations for these subscales using the modified Pearson-Filon statistic [32].

The final hypothesis (that higher-order feeding dimensions and styles would show higher levels of stability over meals and over time) was tested by comparing the stability of demandingness, responsiveness, and feeding style (the three higher-order constructs) with the stability of the individual feeding practices. For across meal comparisons, this involved comparing the mean correlations for the higher-order constructs to the mean correlations for the individual behaviors. As above, a 0.20 difference between correlations was considered evidence of a difference in stability. For the across time comparisons, the Pearson-Filon statistic was used to compare the correlations for the higher-order constructs over time to the across-time correlations for the individual behaviors.

## Results

Given the rather large number of findings presented here, we present the results by hypothesis.

### Hypothesis 1: Stability of feeding practices and styles across meals

The first hypothesis predicted that mothers would show moderate levels of stability in their feeding practices and styles across meals within a time point. Presented in Table 3 are the average correlations across the three meals for the observed feeding practices. As can be seen in the table, only encourage eating (.62), spoon feeding (.54), and non-influence attempts about the target child (.63) showed high levels of stability across meals. All but one of the other desired behavior categories showed moderate levels of stability (correlations ranging from .33 to .47). Influence attempts involving non-food related behaviors showed a low level of stability (.23), although this level of stability was still significantly significant. Stability of the strategies was somewhat lower than the desired behaviors, with four showing moderate stability—points/motions (.44), unelaborated command (.38), hint/acknowledge (.37), and enthusiastic modeling (.32)—and most of the rest showing low, but statistically significant, stability. For the strategies, only questions/suggestions did not show statistically significant stability across meals (.13). For the non-influence attempts, other food-related attempts and clarification showed moderate stability (.32 and .38 respectively) and references to the mother, food characteristics, and non-food related content showed low stability (.21, .19, and .23 respectively). References to other people (.09) were not statistically significant.

**Table 3:** Stability of Observed Feeding Behaviors across Three Meals (Study 1) and Across 18 Months (Study 2) (Codes with Low Frequencies Not Reported)

	Mean (SD) Across 3 Meals	Mean Correlation across 3 Meals^a^	Mean (SD) Across 2 Timepoints	Correlation across 2 Time points (18 Months)^b^
Frequency of Influence Attempts (Desired Behaviors)				
Encourage Eating	20.44 (21.75)	**.62**	34.94 (23.06)	**.39**
Eat All Food on Plate	1.48 (2.63)	**.39**	5.91 (5.48)	**.19**
Eat a Different Food	3.73 (4.10)	**.40**	38.53 (18.26)	**.46**
Discourage Eating	2.31 (2.47)	**.35**	0.07 (.04)	**.35**
Table Manners	7.52 (8.66)	**.47**	17.12 (12.42)	**.45**
Eating Skills	3.02 (3.33)	**.33**	6.48 (5.10)	**.22**
Other Food Related Behaviors	1.62 (2.71)	**.41**	13.69 (10.84)	**.51**
Non-Food Related Behaviors	1.68 (2.59)	**.23**	2.81 (3.54)	.08
Proportion of Influence Attempts (Verbal Strategies)				
Hint/Acknowledge	0.05 (0.07)	**.37**	0.06 (0.04)	.08
Enthusiastic Modeling	0.02 (0.03)	**.32**	0.08 ((0.05)	**.49**
Question/Suggestion	0.18 (0.12)	.13	0.26 (0.08)	**.28**
Reason/Instruct	0.07 (0.05)	**.22**	0.06 (0.04)	**.23**
Unelaborated Command	0.52 (0.15)	**.38**	0.40 (0.10)	**.39**
Verbal Pressure	0.02 (0.03)	**.26**	0.01 (0.01)	−.06
Proportion of Influence Attempts (Nonverbal Strategies)				
Moves Self Closer	0.03 (0.05)	**.22**	0.01 (0.01)	.14
Moves Something Closer	0.06 (0.06)	**.21**	0.04 (0.02)	.11
Points/Motions	0.17 (0.13)	**.44**	0.30 (0.09)	**.23**
Helps	0.04 (0.05)	**.20**	0.01 (0.01)	−.12
Spoon Feeds	0.03 (0.06)	**.54**	0.02 (0.02)	**.27**
Physically Forces	0.03 (0.04)	**.23**	0.01 (0.01)	.11
Frequency of Non-Influence Attempts^c^				
Food Characteristics	1.30 (2.12)	**.19**	–	–
Other-Food Related	0.65 (1.30)	**.32**	–	–
Target Child	5.58 (7.16)	**.63**	–	–
Mother	0.54 (0.91)	**.21**	–	–
Other People	0.87 (1.60)	.09	–	–
Non-Food Related	3.94 (5.62)	**.23**	–	–
Clarification	1.96 (2.76)	**.38**	–	–
Feeding Dimensions				
Demandingness (rate/min)	1.31 (1.19)	**.63**	1.00 (0.57)	**.34**
Responsiveness (proportion child strategies)	0.35 (0.15)	**.33**	0.50 (.11)	**.49**
Feeding Styles (1 = yes, 0 = no)				
Authoritative	**–**	**.22**		**.33**
Authoritarian	**–**	**.23**		**.30**
Indulgent	**–**	**.21**		**.19**
Uninvolved^c^	**–**	**.21**		.02

^a^correlations ≥ .18 (bolded) were significant at the *p* < .05 level

^b^correlations ≥ .17 (bolded) were significant at the *p* < .05 level

^c^Coded in Study 1 only

Regarding the observational measures of feeding dimensions and styles (see Table 3), demandingness showed higher levels of stability (.63) than responsiveness (.33) and the four feeding styles showed low, but statistically significant levels of stability (.21 to .23). Mothers had the same feeding style across pairs of meals about 40% of the time—44% for authoritative, 42% for uninvolved, 41% for authoritarian, and 39% for indulgent.

Overall, this hypothesis was supported, especially for the influence attempts, strategies, and feeding styles. With the exception of talking about the target child and clarification, non-influence attempts showed less stability.

### Hypothesis 2: Stability across 18 Months versus within a time point

The second hypothesis was that mothers would show greater levels of stability of observed behavior across meals than across 18 months. Presented in Table 3 are the correlations of the observational variables across 18 months. As was the case for the within time point correlations, for the desired behaviors and verbal strategies, most of the correlations were moderate in size (one high, six moderate, four low, and three nonsignificant). Only two of the nonverbal strategies were significant, and both of these were low—spoon feeds (.27) and points motions (.23).

For the desired behaviors, comparing the two columns in Table 3 code by code shows that in most cases the level of stability over time and across meals was similar. Only two correlations for the desired behaviors differed by .20 or more—encourage eating and eat all food on the plate showed higher stability across three meals than across 18 months For the strategies, five correlations differed by .20 or more and in each case the correlation was higher across three meals than over time—i.e., hint/acknowledge, verbal pressure, points/motions, helps, and spoon feeds.

Regarding the observed feeding dimensions and styles, responsiveness (.49) showed slightly higher levels of stability over 18 months than demandingness (.34) (both were moderately stable). Demandingness showed greater stability across meals (.63) than over time (.34). All except the uninvolved style showed statistically significant stability over time. Stability of the authoritative (.33) and authoritarian (.30) styles were moderate and higher than the stability of the indulgent style (.19) (see Table 3). The authoritative and authoritarian feeding styles were stable across time points about half the time (48% and 51% respectively), the indulgent style was stable 44% of the time, and the uninvolved style stable only 22% of the time. The stability of the feeding styles was not different across meals than over time.

Overall, hypothesis 2 was supported as well. Although some maternal behaviors did not differ in stability in the comparison across meals and over time, eight differences were found, all in the predicted direction. As for feeding styles, the levels of stability did not differ.

### Hypothesis 3: Self-reports versus observed feeding over time

Hypothesis 3 predicted that self-reports of feeding would be more stable over 18 months than the observed feeding variables. Presented in Table 4 are the time 1 – time 2 correlations for the subscales of the various parent feeding questionnaires in Study 2. Correlations were only calculated for subscales with coefficient alphas greater than or equal to .60. As can be seen in the table, with the exception of the monitoring subscales in the CFQ and the CFPQ that showed low stability (where the across time correlations were .19 and .29 respectively), the across time correlations for the CFQ and CFPQ ranged from .38 to .66 (mean = .50). Five of the correlations for the individual subscales were in the high stability range: restriction for weight purposes (.66), pressure to eat (.57 and .60), child control (.50), and involvement (.50). The demandingness and responsiveness dimensions on the CFSQ were in high range as well (.62 and .51 respectively). The remaining five subscales were in the moderate range: modeling (.49), emotion regulation (.46), restriction (CFQ) (.44), restriction for health reasons (.40) and balance and variety (.38). Examination of the stability of the four CFSQ feeding styles over 18 months (not shown in the table) was much lower: authoritative (*r* = .24, *p* < .01), authoritarian (*r* = .38, *p* < .001), indulgent (*r* = .37, p < .001), and uninvolved (*r* = .17, *p* = .05). Stability of the CFSQ feeding style across the two time points was greater for the authoritarian (60%) and indulgent (56%) styles than for the authoritative (39%) and uninvolved (29%) styles.

**Table 4:** Coefficient Alphas and Correlations across 18 Months for the Feeding Questionnaire Subscales for Study 2 (*n* = 138)

Questionnaire and Subscale	Number of Items	Coefficient Alpha (Time 1)	Time 1- Time 2 Correlation
Child Feeding Questionnaire (CFQ)			
Restriction	8	.70	.44***
Pressure to Eat	4	.60	.57***
Monitoring	3	.84	.19*
Comprehensive Feeding Practices Questionnaire (CFPQ)			
Child Control	5	.63	.50***
Emotion Regulation	3	.80	.46***
Balance & Variety	4	.67	.38***
Environment	4	.56	–
Food as Reward	3	.50	–
Involvement	3	.70	.50***
Modeling	4	.79	.49***
Monitoring	4	.86	.29***
Pressure	4	.61	.60***
Restriction-Health	4	.63	.40***
Restriction-Weight	8	.83	.66***
Teaching Nutrition	3	.29	–
Caregiver Feeding Style Questionnaire (CFSQ)			
Demandingness	19	na	.62***
Responsiveness	19	na	.51***

*p < .05, **p < .01, ***p < .001

A comparison of the mean correlations across time for the sets of observational and self-report variables that measured similar constructs (see data analysis section), showed that the mean correlation was .36 for the observational measures (moderate stability) and .53 for the self-report measures (high stability).

As shown in Table 3, the observational measures of demandingness and responsiveness showed moderate stability across time (.34 and .49 respectively), whereas the self-reports (see Table 4) showed high levels of stability (.62 and .51). The difference between the demandingness correlations was significant at *p* < .01.

Overall, the pattern of correlations was consistent with the third hypothesis that greater stability would be found for the self-report than the observational measures.

### Hypothesis 4: Differential stability of the self-report subscales

The fourth hypothesis stated that maternal reports of pressure to eat and restriction would show greater levels of stability over an 18-month period than maternal monitoring. Examination of Table 4 shows that this hypothesis was supported—as mentioned above, the time 1- time 2 correlations for the two monitoring subscales were much lower than the correlations for the remaining self-report subscales. These differences were significant at *p* < .01.

### Hypothesis 5: Stability of higher-order measures versus individual feeding practices

The final hypothesis proposed that for both observed and self-report measures, the higher-order measures of feeding dimensions and styles (i.e., measures that aggregate across multiple individual feeding behaviors) would show higher levels of stability over time and across meals than individual feeding practices. This hypothesis was tested by comparing the stability of demandingness, responsiveness, and feeding style (the three higher-order constructs) with the stability of the individual feeding practices. For the observational data, the hypothesis was partially supported for demandingness—the stability of this measure across meals was among the highest values (.63). In contrast, across 18 months, it only showed moderate levels of stability (.34) that differed little from many of the individual behaviors. The converse was true for observed responsiveness—it showed a level of stability that was among the highest across 18 months (.49), but the stability across meals was moderate (.33) and similar to many individual behaviors. Finally, the stability of feeding styles was low—much lower than many of the individual behaviors. Therefore, there was only partial support for this hypothesis.

## Discussion

The purpose of this study was to examine consistency in maternal feeding practices and styles across situations and over time. Together the results showed that there was only moderate stability across meals and over 18 months for most of the observational and self-report measures. Moreover, the levels of the stability varied depending on the nature of the feeding behavior being studied. The results were largely consistent with the hypotheses.

Although both the desired behavior and strategy codes showed moderate levels of stability across meals and over time, the stability of the strategy codes was somewhat lower. This suggests that although mothers may be moderately consistent in their feeding goals (e.g., encouraging eating, discouraging eating, enforcing table manners), they may be more flexible in the strategies that they use to achievement them on any particular day. With a couple of exceptions (e.g., a consistent tendency to talk or not talk about the target child), the non-influence attempts were less consistent across meals and time than the influence attempts. Because non-influence attempts tended to be general conversation between the mother and child, it is not that surprising that conversational content varied more across meals than maternal directives to eat or not eat.

The moderate stability of both the observational and self-report measures suggests that both approaches to measuring feeding help identify moderately consistent individual differences between mothers in their feeding practices and styles. However, because only moderate levels of stability were observed, feeding behavior varied considerably across situations. The lack of high levels of consistency across meals and over time suggests that situational factors may contribute significantly to individual differences in maternal feeding practices as well. A lack of consistency across meals or over time could reflect random patterns of variation or systematic variation attributed to specific situational factors. Possible situational factors might include: the nature of mother-child interactions earlier in the day or at previous meals, mother or child emotional state, the time of day, the food served, the length of the meal, the presence of distractions, and so on. Future research should directly assess the impact of situational factors on feeding practices in order to identify those factors that account for important differences in parental feeding practices and styles. Such factors could then be targeted in interventions to improve parental feeding.

Direct comparisons of stability across meals and over time is difficult in this paper because we compared the results of two studies that differed in their methods—long-term stability was assessed in a laboratory setting with the same food at each time point, whereas short-term stability was assessed at home during three different meals prepared by the mother. These differences in the situations and the foods served, however, may have reduced the size of the differences we observed between the two sets of correlations. That is, the situational factors in the controlled laboratory study at the two time points were likely more similar than the situational variations in the less controlled study of three meals at home. The situational similarities in the lab study, therefore, might have inflated the stability of maternal behaviors over 18 months when compared to the three-meal study. Despite these differences, however, greater stability was found for a number of behaviors across meals than across 18 months. Given the significant changes in children’s development and experiences over this relatively long time period, these results are not that surprising. What *is* surprising, however, is that a number of behaviors showed similar levels of consistency across meals and over 18 months. Examination of these behaviors help identify several individual differences in maternal feeding practices that appear to remain fairly consistent over time and situations—i.e., the tendency to discourage or redirect eating (discourage eating and eat a different food), the tendency to enforce table manners and teach eating skills, the use of enthusiastic modeling, and the use of reasoning versus unelaborated commands. Feeding styles also showed similar levels of consistency across meals and over time. This may be the result of the fact that feeding styles represent individual differences in general feeding styles aggregated across situational variation.

Consistent with previous studies [16, 17], with the exception of monitoring, the self-reports of feeding practices were moderately to highly stable. The observational measures showed less stability than the self-reports. As Mischel [18] argued, self-reports may more likely reflect people’s cognitive constructions of what their behavior is like rather than their actual behavior. This could mean that mothers might have completed the questionnaire in terms of the kind of mother they thought they were, presenting themselves in a particular way. These conceptions are likely more stable over time than observed maternal behaviors that can vary considerably across situations and time.

The hypothesis receiving the least empirical support was that the higher-order measures of feeding dimensions and styles would show higher levels of stability over meals and over time than individual feeding practices. For the two higher-order feeding dimensions (demandingness and responsiveness) support for this hypothesis was mixed. Furthermore, the hypothesis was not supported for feeding styles. One possible reason for the lower consistency of feeding styles is that by dichotomizing demandingness and responsiveness to yield the measure of feeding styles, meaningful variation in these measures may have been lost. In the future, researchers should work to identify other higher-order feeding constructs that show greater levels of stability across situations and over time than those studied here.

The results of this study should be considered within its limitations. First, the generalizability of the findings is limited to low-income Latina and African-American mothers. Mothers with different ethnic and socioeconomic backgrounds may differ in the stability of their feeding behaviors across meals and across time. Future research should explore these differences, as well as some of the factors that account for any differences found. Second, social desirability likely influenced both the observational and self-reported measures because mothers might have wanted to portray themselves in a positive way. Third, the findings for demandingness may have been more valid than the findings for responsiveness. The measurement of demandingness was a direct measure because it measured every time the mother was trying to get the child to eat more food. The measure of responsiveness, in contrast, was a more indirect measure in that responsiveness was inferred from the use of less directive versus more directive maternal feeding strategies. Individual differences in the *quality* of these strategies (e.g., tone of voice, responsiveness to children’s cues) would likely have provided a more accurate measure of his construct.

Despite these limitations, there were a number of strengths of this study that help it contribute to the literature in this area. First, these were large samples with sufficient power for most analyses. Second, the use of both self-report and observational measures of feeding allowed for a more thorough examination of maternal feeding behavior. Third, this study, to our knowledge, is the only study to use observational measures to examine the feeding styles identified by Hughes and colleagues [11] in their work with the CFSQ questionnaire. Finally, this is the first study to examine stability and change in observed feeding behavior across meals and across time.

## Conclusions

This study has begun to fill in the gap in the literature to better understand parental feeding practices, dimensions, and styles as assessed through observations and self-reports. It is crucial that future research consider how situational variables play a role in maternal feeding. For example, within-subject designs could examine how maternal mood affects mothers’ behavior during mealtime or how the child’s behavior during the meal impacts the mothers’ feeding behavior. Situational factors should be taken into account in designing prevention/intervention programs as well. For example, prevention programs might help mothers avoid rushing when feeding their children or help mothers learn how structured mealtime routines can increase their children’s interest in trying new foods. Overall, this study examined Latina and African American mother’s feeding interactions with their children using observational and self-report data. Studies of other populations should be conducted as well to further understand the development of parental feeding practices, dimensions, and styles and the prevention of childhood obesity.

## Abbreviations

BMI: Body mass index
CFPQ: Comprehensive feeding practices questionnaire
CFQ: Child feeding questionnaire
CFSQ: Caregiver’s feeding style questionnaire
